# Base Editing Mediated Generation of Point Mutations Into Human Pluripotent Stem Cells for Modeling Disease

**DOI:** 10.3389/fcell.2020.590581

**Published:** 2020-09-25

**Authors:** Tao Qi, Fujian Wu, Yuquan Xie, Siqi Gao, Miaomiao Li, Jun Pu, Dali Li, Feng Lan, Yongming Wang

**Affiliations:** ^1^State Key Laboratory of Genetic Engineering, School of Life Sciences, Zhongshan Hospital, Fudan University, Shanghai, China; ^2^State Key Laboratory of Cardiovascular Disease, Fuwai Hospital, National Center for Cardiovascular Diseases, Chinese Academy of Medical Sciences and Peking Union Medical College, Beijing, China; ^3^Department of Cardiology, Renji Hospital, School of Medicine, Shanghai Jiao Tong University, Shanghai, China; ^4^Shanghai Key Laboratory of Regulatory Biology, Institute of Biomedical Sciences and School of Life Sciences, East China Normal University, Shanghai, China; ^5^Shanghai Engineering Research Center of Industrial Microorganisms, Shanghai, China

**Keywords:** human pluripotent stem cell, base editing, episomal vector, disease modeling, IPS, long QT syndrome, Brugada syndrome

## Abstract

Human pluripotent stem cells (hPSCs) are a powerful platform for disease modeling and drug discovery. However, the introduction of known pathogenic mutations into hPSCs is a time-consuming and labor-intensive process. Base editing is a newly developed technology that enables facile introduction of point mutations into specific loci within the genome of living cells. Here, we design an all-in-one episomal vector that expresses a single guide RNA (sgRNA) with an adenine base editor (ABE) or a cytosine base editor (CBE). Both ABE and CBE can efficiently introduce mutations into cells, A-to-G and C-to-T, respectively. We introduce disease-specific mutations of long QT syndrome into hPSCs to model LQT1, LQT2, and LQT3. Electrophysiological analysis of hPSC-derived cardiomyocytes (hPSC-CMs) using multi-electrode arrays (MEAs) reveals that edited hPSC-CMs display significant increases in duration of the action potential. Finally, we introduce the novel Brugada syndrome-associated mutation into hPSCs, demonstrating that this mutation can cause abnormal electrophysiology. Our study demonstrates that episomal encoded base editors (epi-BEs) can efficiently generate mutation-specific disease hPSC models.

## Introduction

Human pluripotent stem cells (hPSCs), including human embryonic stem cells (hESCs) (Thomson et al., 1998) and the closely related human induced pluripotent stem cells (iPSCs) (Takahashi et al., 2007), can self-renew and differentiate into various cell types. Due to these properties, these stem cells hold great promise for modeling mammalian organ development, studying disease mechanisms and pathways, and developing future therapies (Wang et al., 2012, 2014; Lan et al., 2013; Li et al., 2019). However, to realize these potential applications, genes of interest often need to be engineered in the cell. For example, iPSCs derived from patients often contain genetic mutations that need to be corrected prior to conducting specific therapies (Maeder and Gersbach, 2016). For disease modeling with normal hPSCs, pathogenic mutations need to be introduced into cells to generate disease models (Wang et al., 2014; Xie et al., 2019). Therefore, it is crucial to develop methods and technologies for efficient genetic manipulation of stem cells.

The RNA-guided CRISPR–Cas9 system is a powerful tool for genome editing in hPSCs (Mali et al., 2013; Xie et al., 2017). In this system, a Cas9 nuclease and a single guide RNA (sgRNA) form a Cas9–sgRNA complex, which recognizes a specific DNA sequence that is complementary to the sgRNA, and subsequently generate a site-specific double-strand break (DSB) (Jinek et al., 2012; Cong et al., 2013; Mali et al., 2013). The DSBs are typically repaired by the cell’s endogenous DNA repair machinery through non-homologous end-joining (NHEJ) or microhomology mediated end-joining (MMEJ), resulting in non-specific small insertions and deletions (indels) around the cut site, which can be useful for generating loss-of-function mutations (Kim and Kim, 2014). These DSBs can also be repaired by homology-directed repair (HDR) using an introduced DNA repair template, such as a double-stranded DNA donor plasmid or single-stranded donor DNA oligonucleotides (ssODN), resulting in the introduction of precise modifications (Wang et al., 2012; Kim and Kim, 2014). However, the HDR pathway is strongly disfavored in most cell types and requires special cellular machinery that is only present during cellular replication (Wang et al., 2012, 2014). Furthermore, the use of nuclease Cas9 to mediate DSBs results in potential undesired translocations or DNA rearrangements.

Base editing is a new genome-editing technology that directly generates precise point mutations without generating DSBs (Komor et al., 2016; Gaudelli et al., 2017). Base editors comprise fusions between a catalytically impaired Cas9 nuclease and a single-stranded DNA (ssDNA) specific deaminase enzyme. Upon binding to its target DNA, base pairing between the sgRNA and the target DNA strand results in the displacement of a small segment of single-strand DNA as an “R-loop” (Nishimasu et al., 2014; Rees and Liu, 2018). DNA bases within this ssDNA are therefore substrates for deamination and are subsequently modified by the deaminase enzyme. Two classes of DNA base editor have so far been developed—cytosine base editors (CBEs) convert a C⋅G base pair into a T⋅A base pair (Komor et al., 2016), and adenine base editors (ABEs) convert an A⋅T base pair into a G⋅C base pair (Gaudelli et al., 2017). Base editors hold great promise for the genetic modification of hPSCs (Chadwick et al., 2017).

The long QT syndrome (LQTS) is a heart disorder that is characterized by the prolongation of the QT interval of surface electrocardiograms and is associated with malignant arrhythmias, syncopal episodes, torsade de pointes form ventricular tachycardias, and an increased risk of sudden cardiac death (Moss, 2003). Mutations in the KCNQ1 (LQT1), KCNH2 (LQT2), and SCN5A genes (LQT3) account for the three most common pathogenic variants that are clinically annotated for LQTS (Moss, 2003). In addition to LQTS, mutations in SCN5A have been shown to induce Brugada syndrome (BrS) (Wilde and Amin, 2018). We and others have demonstrated that hPSCs can be used for modeling LQTS (Liang et al., 2013; Wang et al., 2014).

We previously used an episomal vector to express Cas9 and sgRNAs (epiCRISPR), and achieved high efficiency of genome editing through the NHEJ/MMEJ pathway (Xie et al., 2017; Wang et al., 2019). This episomal vector can replicate during cellular division in eukaryotes (Van Craenenbroeck et al., 2000), permitting the continuous expression of Cas9 and sgRNAs in cells. The vector also contains a drug resistance gene, allowing for the enrichment of transfected cells by drug selection. In this study, we generate episomal vectors that express base editors (epi-BEs) and achieve high efficiency of precise genome editing in hPSCs. As a proof of concept, we first demonstrate that these epi-BEs can efficiently introduce mutations into hPSCs to generate LQTS models. We then evaluate a novel mutation on SCN5A for developing BrS. Our study demonstrates that these epi-BEs are a simple and efficient platform to generate human disease models in stem cells.

## Materials and Methods

### Cell Culture

Human ESCs (H9, WA09, Wicell, Madison, WI, United States) and iPSCs were cultured on Matrigel-coated plates (ESC qualified, BD Biosciences, San Diego, CA, United States) using hESC mTeSR-1 cell culture medium (StemCell Technologies, Vancouver, Canada), whereas HEK293T and HeLa cells were maintained in Dulbecco’s Modified Eagle Medium (DMEM) supplemented with 10% fetal bovine serum (FBS, Gibco). All media contained 100 U/ml of penicillin and 100 mg/ml of streptomycin. Cells were cultured at 37°C with 5% CO_2_ in a humidified and mycoplasma negative incubator.

### Plasmid Construction

Epi-ABEmax/epi-AncBE4max: KO plasmid (Addgene #135970) was PCR-amplified using primers EBV-F/EBV-R to generate Fragment 1, which contained oriP/EBNA1, gRNA expression cassette (hU6 promoter and gRNA scaffold) and EF1A core promoter. Fragment 2 was synthesized by GENEWIZ (Suzhou, China) and was used to express blasticidin S deaminase (BSD), and then Fragment 2 was PCR-amplified using primers BSD-F/BSD-R. Fragment 3 contained full ABE or CBE and was obtained from pCMV_ABEmax (Addgene #112095) or pCMV_AncBE4max (Addgene #112094); this was amplified by PCR using primers ABEmax-F/ABEmax-R or AncBE4-F/AncBE4-R. All three fragments were cloned together by NEBuilder Assembly Tool (NEB) following the manufacturer’s protocol, resulting in epi-ABEmax-hU6 or epiAncBE4-hU6. Furthermore, hU6 was replaced by mU6 to eventually result in epi-ABEmax or epi-AncBE4max. mU6 was synthesized by GENEWIZ (Suzhou, China) and PCR-amplified using primers mU6-F/mU6-R, then cloned into the epi-ABEmax-hU6 or epi-AncBE4max-hU6 backbone, which was PCR-amplified using primers EBV-2-F/EBV-2-R. Sequences were verified by Sanger sequencing (GENEWIZ, Suzhou, China).

Epi-ABEmax-NG/epi-AncBE4max-NG: A portion of SpCas9-NG that contained the seven mutations (R1335V/L1111R/D1135V/G1218R/E1219F/A1322R/T1337R) was PCR-amplified from pX330-SpCas9-NG (Addgene #17919) using primers 1111-UP-F/1337-DOWN-R. This portion of SpCas9-NG was cloned into epi-ABEmax or epi-AncBE4max backbone, which was PCR-amplified using primers epi-backbone-F/epi-backbone-R, resulting in epi-ABEmax-NG or epi-AncBE4max-NG.

To express an sgRNA, the oligonucleotide duplexes were cloned into BspQI restriction sites of epi-ABEmax/epi-AncBE4 max/epi-ABEmax-NG/epi-AncBE4max-NG.

All primers are listed in Supplementary Table 1.

### Base Editing and Blasticidin Selection

Cells were plated into 48-well plates and transfected the next day at approximately 70% confluency. Briefly, 500 ng of epi-ABEmax/epi-AncBE4max/epi-ABEmax-NG/epi-AncBE4 max-NG plasmid was transfected using Lipofectamine 3000 (Life Technologies) according to the manufacturer’s instructions at day 1. For H9 and iPSCs, cells were passaged on days 2, 6, and 11, respectively, and selected by blasticidin (Thermo Fisher Scientific) from day 2 to day 16. A 2.5 μg/ml of blasticidin was added into the growth media, except on days 2, 6, and 11, where 10, 5, and 0 μg/ml of blasticidin were used, respectively. For HeLa and HEK293T cells, 2.5 μg/ml of blasticidin was used from day 2 to day 16. Transfected cells were harvested for analysis on days 6, 11, and 16. The antibiotic screening time can be shortened when editing efficiency is enough, which has been discussed in detail in a recent work (Geng et al., 2020).

The genomic DNA was isolated using QuickExtract^TM^ DNA Extraction Solution (Lucigen) according to the manufacturer’s instructions. Targets of base editing were amplified by PCR using KOD DNA Polymerase (Toyobo). The PCR products were sequenced using Sanger sequencing, and the editing efficiency was analyzed by EditR (Kluesner et al., 2018).

### Off-Target Analysis

For each target site, five potential off-targets were selected based on Cas-Offinder^1^ (Bae et al., 2014) and PCR-amplified for Sanger sequencing.

### Single Cell-Derived Clone Screen

The iPSCs were passaged with ethylenediaminetetraacetic acid (EDTA). Then, 1 × 10^5^ cells were seeded on a Matrigel (Corning, United States) pre-coated 10 cm dish using E8 media (Cellapy, Beijing, China) with 2 μM of thiazovivin (TZV, Selleck, United States). Twenty-four hours later, the E8 media was replaced by new media without TZV. This media was changed every 2 days. Ten days after seeding, the single cell-derived clones were picked with a 1 ml sterile syringe and placed on a Matrigel pre-coated 24-well plate for cell expansion. Genomic DNA was extracted using the TIANamp Genomic DNA Kit (Tiangen Biotech, Beijing, China) for DNA sequencing.

### Cardiomyocyte Differentiation

A chemically defined molecular-based method was applied for cardiomyocyte (CM) differentiation (Burridge et al., 2015). Briefly, cells (∼90% confluency) were seeded on a Matrigel pre-coated 6-well plate at a ratio of 1:6 in E8 media. The media was changed to CDM3 (consisted of RPMI 1640, 500 μg/ml of *Oryza sativa*-derived recombinant human albumin, and 213 μg/ml of L-ascorbic acid 2-phosphate) supplemented with 6 μM of CHIR99021 when the cells reached ∼75% confluency. After 48 h, the media was changed to CDM3 supplemented with 2 μM of Wnt-C59. After 2 days, the media was changed to CDM3 and refreshed every 2 days. After differentiation for 7–8 days, spontaneous contracting cells could be observed. On day 12, CMs were purified using a metabolic-selection method. Briefly, the medium consisted of RPMI 1640 without glucose, 213 μg/ml of L-ascorbic acid 2-phosphate, 500 μg/ml of *Oryza sativa*-derived recombinant human albumin, and 5 mM of sodium DL-lactate (Sigma, United States). After purification, CMs were cultured with RPMI 1640 and B27 (Life Technology, United States). For cellular maintenance, the medium was changed every 3 days.

### Electrophysiological Test Using MEA

CMs were digested with Accutase (Thermo Fisher, United States). CytoView MEA24 plates (Axion Biosystems, Inc., Atlanta, United States) were pre-coated overnight by 0.5% Matrigel phosphate-buffered saline (PBS) solution. Then, 15,000 CMs were plated on each multi-electrode array (MEA) well with RPMI/B27 medium and cultured for 3 days. When the cellular electrophysiological activity became stable, the experimental data were recorded using Maestro EDGE (Axion Biosystems, Inc., Atlanta, United States) according to the MEA manual. The data were analyzed using the AxIS Navigator, Cardiac Analysis Tool, and IGOR software.

### Clinical Data

The patient’s data were provided by Xin Hua Hospital Affiliated to Shanghai Jiao Tong University.

### Quantification and Statistical Analysis

The editing efficiency was analyzed by EditR, which is an algorithm for predicting potential editing in a guide RNA region from a single Sanger sequencing run (Kluesner et al., 2018). All data are shown as mean ± SD. Statistical analyses were conducted using Microsoft Excel. Two-tailed, unpaired Student’s *t*-tests were used to determine statistical significance when comparing two groups. A value of *P* < 0.05 was considered to be statistically significant (^∗^*P* < 0.05, *^∗∗^P* < 0.01, *^∗∗∗^P* < 0.001, ^****^P < 0.0001).

## Results

### Episomal Vector-Based Base Editors for Efficient Base Editing

**FIGURE 1:**
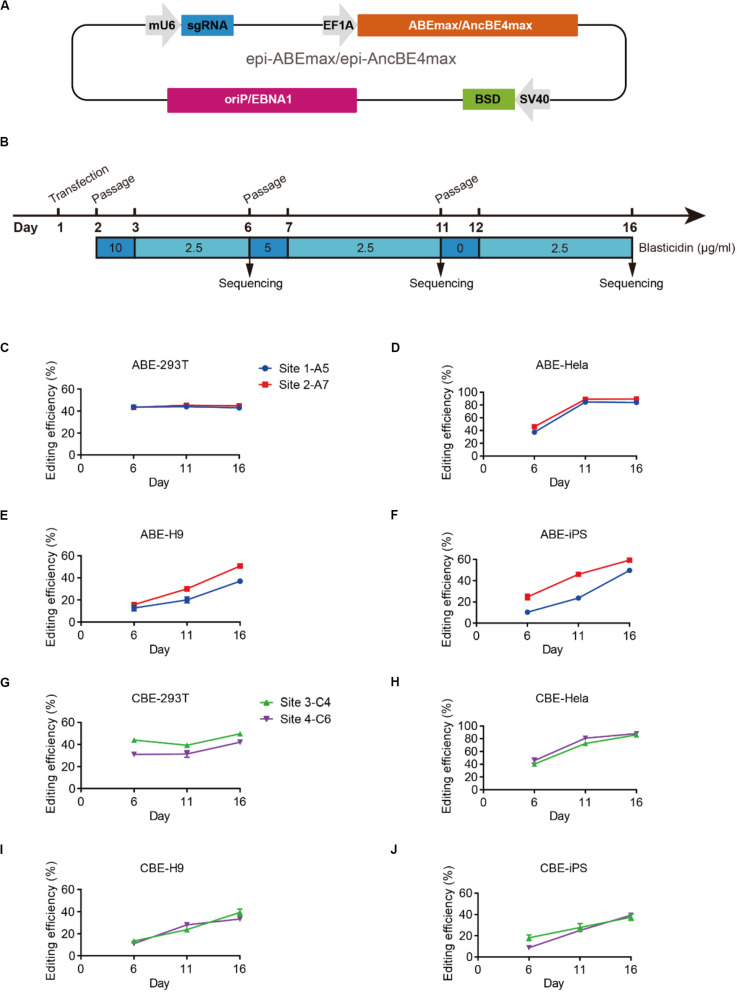
Efficient base editing with epi-ABEmax and epi-AncBE4max. **(A)** Schematic of the epi-ABEmax and epi-AncBE4max plasmids. The vector contains a mouse U6 promoter-driven gRNA scaffold, an EF1a promoter-driven base editor, an SV40 promoter-driven a blasticidin S deaminase (BSD), and oriP/EBNA1 elements for the plasmid replication in eukaryotes. **(B)** Procedure of base editing with epi-ABEmax and epi-AncBE4max. **(C–F)** Editing efficiency of epi-ABEmax in HEK293T, HeLa, H9, and iPSCs was measured at three-time points, *n* = 3 independent experiments. **(G–J)** Editing efficiency of epi-AncBE4max in HEK293T, HeLa, H9, and iPSCs was measured at three-time points, *n* = 3 independent experiments.

We designed an all-in-one episomal vector-based system to express an sgRNA with an ABE (ABEmax, Koblan et al., 2018), which we named epi-ABEmax, or a CBE (AncBE4max, Koblan et al., 2018), which we named epi-AncBE4max. These vectors also express a BSD conferring resistance to blasticidin, which allows for enrichment of transfected cells through antibiotic selection (Figure 1A). First, we analyzed the capacity of the epi-ABEmax for base editing in two somatic cell lines (HEK293T and HeLa) and two hPSC lines (H9 and iPSCs). We selected two previously reported sgRNAs, targeting Site 1 and Site 2 (Koblan et al., 2018), to determine adenine base editing efficiency. The plasmid was delivered into cells using a lipid-based transfection, followed by blasticidin selection (Figure 1B). Editing efficiency was measured on days 6, 11, and 16. The maximum editing efficiencies were observed on days 6 and 11 for HEK293T and HeLa cells (Figures 1C,D). For H9 and iPSCs, the editing efficiency increased over time and seems to continue growing past day 16 (Figures 1E,F).

Next, we analyzed the capacity of the epi-AncBE4max for base editing in these four cell lines. We selected two gRNAs, targeting HEK3 (Site 3) (Komor et al., 2016) and FANCF (Site 4), to measure cytosine base editing efficiency. Similar to epi-ABEmax data, the maximum editing efficiencies were observed at days 6 and 11 for HEK293T and HeLa cells (Figures 1G,H). For H9 and iPSCs, the editing efficiency increased over time and seems to continue to increase past day 16 (Figures 1I,J). In summary, these epi-BEs enable efficient base editing in both somatic cells and hPSCs.

### Base Editing of KCNQ1 for LQT1 Modeling

**FIGURE 2:**
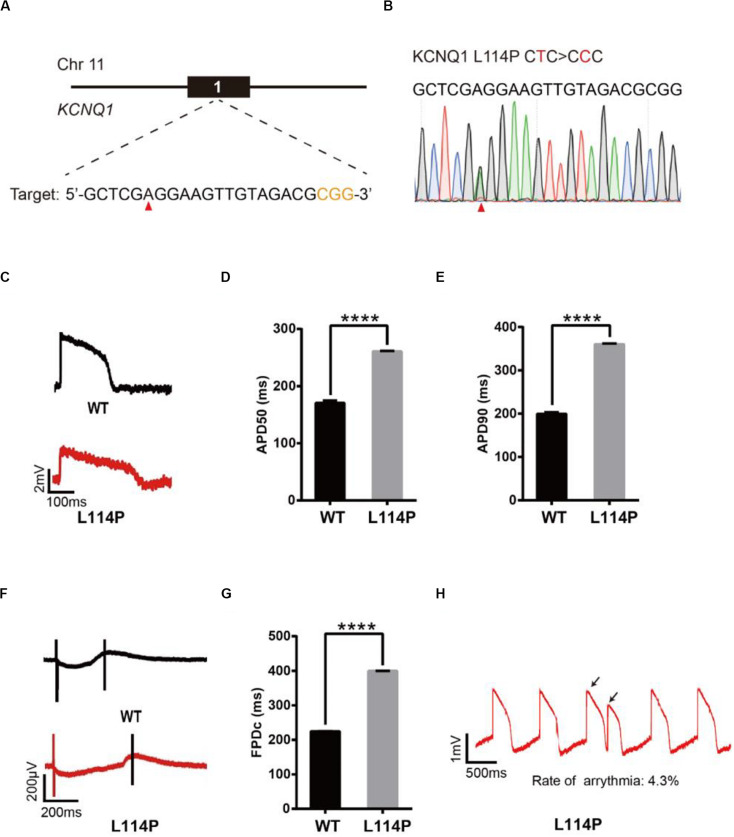
Base editing of L114P-KCNQ1 for LQT1 modeling. **(A)** L114P target site on KCNQ1. “CGG” PAM sequence is shown in orange; target nucleotide to be edited is indicated by a red triangle. **(B)** A heterozygous clone is confirmed by Sanger sequencing. The mutated nucleotide is shown in red; the mutated nucleotide is indicated by a red triangle. **(C)** Single trace of action potentials in WT-CMs and L114P-CMs. **(D,E)** Quantification of action potential at APD50 and APD90. *n* = 3 independent experiments, unpaired *t*-test, *P* < 0.0001. **(F)** Signals of field potential duration recorded by MEA. **(G)** Quantification of corrected field potential durations (FPDc). *n* = 3 independent experiments, unpaired *t*-test, *P* < 0.0001. **(H)** Representative traces of action potentials. The abnormal AP signals were labeled by black arrows. A value of *P* < 0.05 was considered to be statistically significant (*P < 0.05, **P < 0.01, ***P < 0.001, ****P < 0.0001).

We next decided to show the utility of epi-BEs for modeling disease. For this, we introduced the pathogenic mutation L114P in KCNQ1, as annotated in previous literature (Jongbloed et al., 2002), into H9 cells. We designed a corresponding sgRNA (Figure 2A) that would install this mutation located at position 6 of the protospacer, counting the SpCas9 PAM as positions 21–23. Following a similar editing protocol as before, the editing efficiency reached up to 34% on day 16 (Supplementary Table 2 and Supplementary Figure 1A). We screened 52 single cell-derived clones, from which there were 26 heterozygous clones and 3 homozygous clones (Table 1). To test whether base editing influenced the pluripotency of H9 cells, immunofluorescence staining was performed for the pluripotency markers OCT-4 and SSEA-4. Besides, a higher standard, teratoma formation experiment was performed. We note that base editing did not influence marker expression, and that the cells can differentiate into three germ layer lineages after base editing (Supplementary Figure 2).

**TABLE 1:** Summary of base editing efficiency.

Gene	Mutation	Total number of clones	Heterozygous clones	Homozygous clones
KCNQ1	L114P	52	26 (50%)	3 (5.8%)
KCNQ1	R190Q	77	22 (28.6%)	6 (7.8%)
KCNH2	Y616C	4	4 (100%)	0 (0)
KCNH2	Y475C	41	18 (40.9%)	8 (19.5%)
SCN5A	E1784K	107	32 (29.9%)	5 (4.7%)
SCN5A	R1879W	47	2 (4.3%)	8 (17%)

Subsequently, heterozygous L114P-H9 cells were differentiated into CMs using a chemically defined, small molecule-based monolayer CM differentiation method (Figure 2B and Supplementary Figure 2) (Burridge et al., 2014). To test whether these CMs exhibited the LQT1 phenotype, electrophysiology was recorded using the local extracellular action potential (LEAP) assay, which is a high-throughput, non-invasive, label-free, and stable method to measure extracellular field potential (FP) from an intact CM syncytium (Hayes et al., 2019), which can be translated into an action potential (AP) (Hayes et al., 2019). Prolongation of the action potential duration (APD) and field potential duration (FPD) are the most important LQTS electrophysiological phenotypes at the cellular level (Itzhaki et al., 2011; Wang et al., 2014). APD at 50% (APD50) and 90% (APD90) in L114P-CMs were significantly longer than those in wild-type (WT)-CMs (Figures 2C–E). Corrected field potential duration (FPDc) in L114P-CMs was significantly longer than that in WT-CMs (Figures 2F,G). L114P-CMs also exhibited an abnormal AP profile, which resembles a closely coupled single triggered beat (Figure 2H).

To further test the capacity of these epi-BEs, we introduced another known pathogenic mutation (R190Q) (Barsheshet et al., 2012) into KCNQ1 to model LQT1 (Supplementary Figures 3A,B). The editing efficiency reached 31% on day 16 (Supplementary Figure 1B and Supplementary Table 2). We screened 77 single cell-derived clones, of which there were 22 heterozygous clones and 6 homozygous clones (Table 1). We differentiated heterozygous R190Q-H9 cells into CMs and recorded electrophysiology by using the LEAP assay (Supplementary Figures 3C–G). Compared with WT-CMs, R190Q-CMs displayed significantly longer APD and FPDc. R190Q-CMs also exhibited an abnormal AP profile, resembling that of a closely coupled single triggered beat (Supplementary Figure 3H). Taken together, these data demonstrated that both L114P-CMs and R190Q-CMs recapitulated LQT1 phenotype.

### Base Editing of KCNH2 for LQT2 Modeling

**FIGURE 3:**
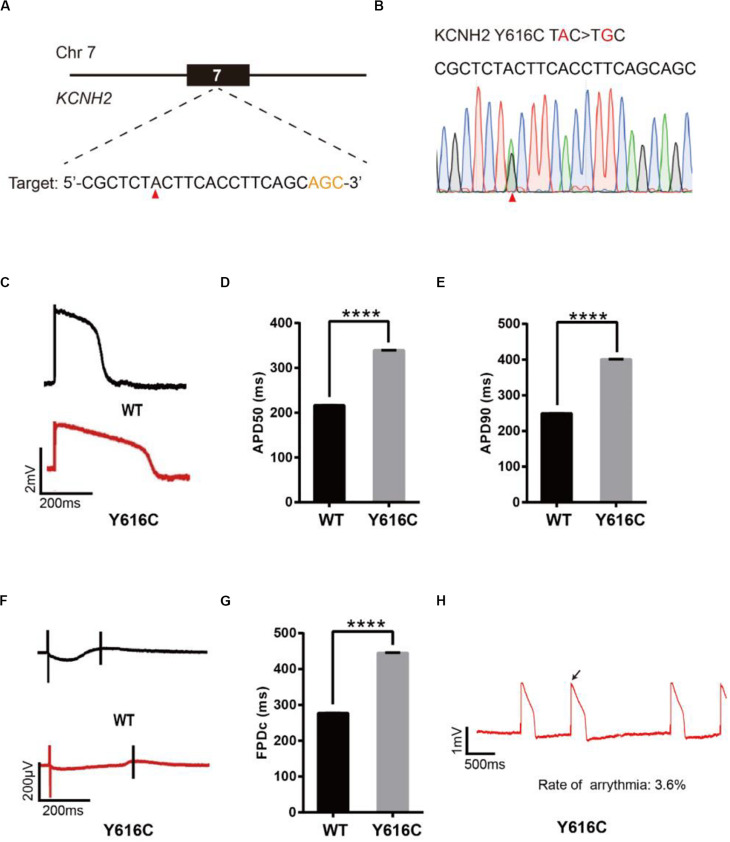
Base editing of Y616C-KCNH2 for LQT2 modeling. **(A)** Y616C target site on KCNH2. “AGC” PAM sequence is shown in orange; target nucleotide is indicated by a red triangle. **(B)** A heterozygous clone is confirmed by Sanger sequencing. The mutated nucleotide is shown in red; the mutated nucleotide is indicated by a red triangle. **(C)** Single trace of action potentials in WT-CMs and Y616C-CMs. **(D,E)** Quantification of action potential at APD50 and APD90. *n* = 3 independent experiments, unpaired *t*-test, *P* < 0.0001. **(F)** Signals of field potential duration recorded by MEA. **(G)** Quantification of corrected field potential durations (FPDc). *n* = 3 independent experiments, unpaired *t*-test, *P* < 0.0001. **(H)** Representative traces of action potentials. The abnormal AP signals were labeled by a black arrow. A value of *P* < 0.05 was considered to be statistically significant (*P < 0.05, **P < 0.01, ***P < 0.001, ****P < 0.0001).

To model LQT2, we introduced a known pathogenic mutation Y616C (Anderson et al., 2014) into the KCNH2 gene of H9 cells (Figure 3A). The editing efficiency reached 37% on day 16 (Supplementary Figure 1C and Supplementary Table 2). We screened four single cell-derived clones, all of which were heterozygous clones (Table 1). To test whether CMs exhibited the LQT2 phenotype, we differentiated the heterozygous Y616C-H9 cells into CMs, and electrophysiology was recorded by the LEAP assay (Figure 3B). APD50 and APD90 in Y616C-CMs were significantly longer than those in WT-CMs (Figures 3C–E). FPDc in Y616C-CMs was significantly longer than that in WT-CMs (Figures 3F,G). Y616C-CMs exhibited an abnormal AP profile, resembling that of a single triggered beat (Figure 3H).

We next introduced another known pathogenic mutation Y475C (Liu et al., 2006) into the KCNH2 gene to model LQT2 (Supplementary Figures 4A,B). We performed editing as described previously and note that the editing efficiency reached 53% on day 16 (Supplementary Figure 1D and Supplementary Table 2). We screened 41 single cell-derived clones, of which there were 18 heterozygous clones and 8 homozygous clones (Table 1). We recorded electrophysiology for the heterozygous Y475C-CMs by LEAP assay (Supplementary Figures 4C–G). Y475C-CMs showed longer APD and FPDc than WT-CMs. Y475C-CMs displayed triggered beats (Supplementary Figure 4H). Taken together, these data demonstrate that both Y616C-CMs and Y475C-CMs recapitulated LQT2 phenotype.

### Base Editing of SCN5A for LQT3 Modeling

**FIGURE 4:**
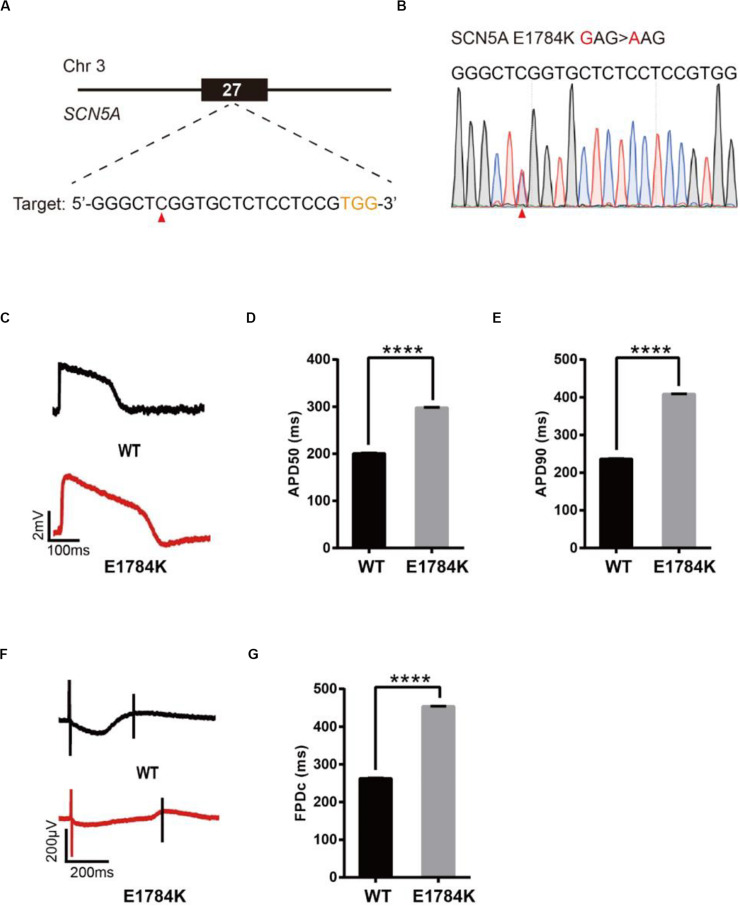
Base editing of E1784K-SCN5A for LQT3 modeling. **(A)** E1784K target site on SCN5A. PAM sequence is shown in orange; target nucleotide is indicated by a red triangle. **(B)** A heterozygous clone is confirmed by Sanger sequencing. The mutated nucleotide is shown in red; the mutated nucleotide is indicated by a red triangle. **(C)** Single trace of action potentials in WT-CMs and E1784K-CMs. **(D,E)** Quantification of action potential at APD50 and APD90. *n* = 3 independent experiments, unpaired *t*-test, *P* < 0.0001. **(F)** Signals of field potential duration recorded by MEA. **(G)** Quantification of corrected field potential durations (FPDc). *n* = 3 independent experiments, unpaired *t*-test, *P* < 0.0001. A value of *P* < 0.05 was considered to be statistically significant (*P < 0.05, **P < 0.01, ***P < 0.001, ****P < 0.0001).

To model LQT3, we introduced a known pathogenic mutation E1784K (Makita et al., 2008) into the SCN5A gene of H9 cells (Figure 4A). The editing efficiency reached 21% on day 16 (Supplementary Figure 1E and Supplementary Table 2). We screened 107 single cell-derived clones, of which there were 32 heterozygous clones and 5 homozygous clones (Table 1). To test whether CMs exhibited the LQT3 phenotype, we differentiated heterozygous E1784K-H9 cells into CMs, and electrophysiology was recorded by the LEAP assay (Figure 4B). APD50 and APD90 in E1784K-CMs were significantly longer than those in WT-CMs (Figures 4C–E). FPDc in E1784K-CMs was significantly longer than that in WT-CMs (Figures 4F,G). These data demonstrated that base-edited E1784K-CMs successfully recapitulated LQT3 phenotype.

### Evaluation of a Novel Mutation for Developing Brugada Syndrome

**FIGURE 5:**
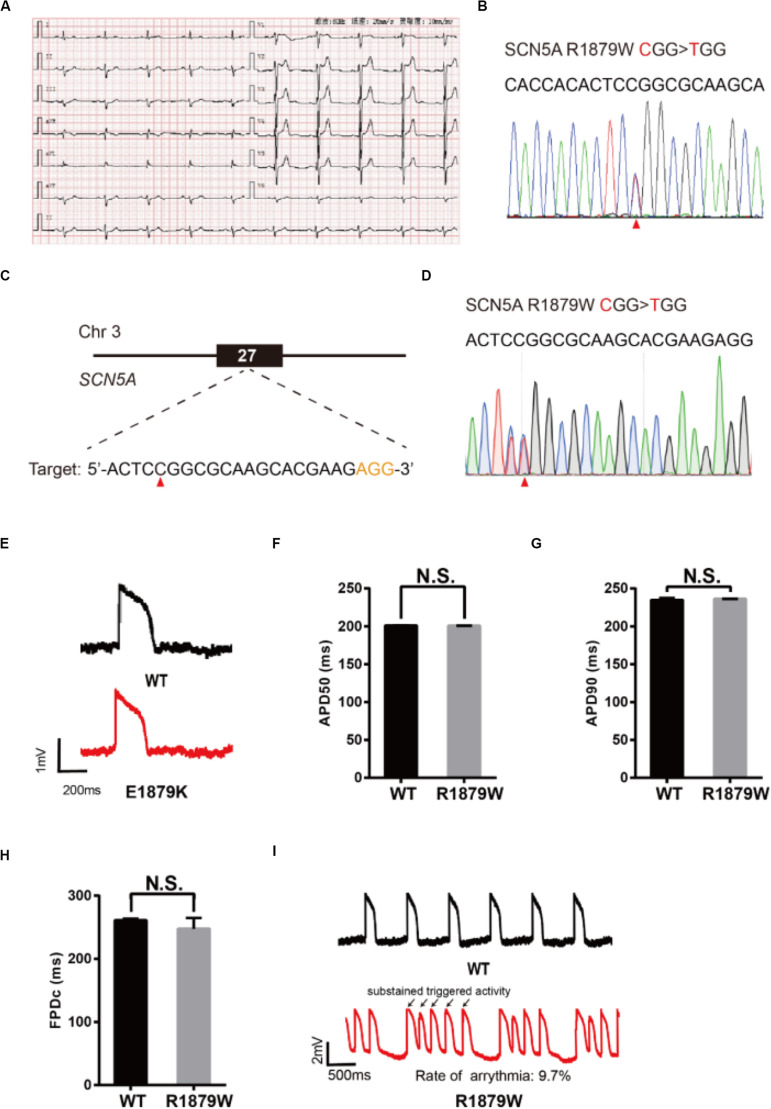
Evaluation of R1879W-SCN5A mutation. **(A)** Patient’s ECG results. The resting ECG showed an ST elevation in leads V1–V3 with a saddle-back pattern. **(B)** Sanger sequencing reveals that a C to T mutation occurs on SCN5A. **(C)** R1879W target site on SCN5A. PAM sequence is shown in orange; target nucleotide is indicated by a red triangle. **(D)** A heterozygous clone is confirmed by Sanger sequencing. The mutated nucleotide is shown in red; the mutated nucleotide is indicated by a red triangle. A neighbor nucleotide C is changed to T, but it does not change the amino acid. **(E)** Single trace of action potentials in WT-CMs and R1879W-CMs. **(F,G)** Quantification of action potential at APD50 and APD90. *n* = 3 independent experiments, unpaired *t*-test. **(H)** Quantification of corrected field potential duration (FPDc). *n* = 3 independent experiments, unpaired *t*-test. **(I)** Representative action potential traces with sustained triggered activity. Triggered beats were labeled by black arrows.

To demonstrate an additional application of epi-BEs, we use it to recapitulate and evaluate a novel mutation identified from a 62-year-old male patient who presented with sudden syncope. The patient’s resting electrocardiogram (ECG) showed an ST elevation in leads V1–V3 with a saddle-back appearance (Figure 5A), which is a characteristic pattern of BrS type 2 (Brugada et al., 2018). Genetic testing revealed a single missense mutation C5635T (R1879W) at the C-terminus of the SCN5A gene (Figure 5B).

To test whether R1879W is a pathogenic mutation, we used the epi-BEs to perform base editing and introduce it into H9 cells (Figure 5C). The editing efficiency reached 18% on day 16 (Supplementary Figure 1F and Supplementary Table 2). We screened 47 single cell-derived clones, of which there were 2 heterozygous clones and 8 homozygous clones (Table 1). To test whether CMs exhibited BrS phenotype, we differentiated heterozygous R1879W-H9 cells into CMs, and electrophysiology was recorded by the LEAP assay (Figure 5D). APD50, APD90, and FPDc of R1879W-CMs showed no significant difference compared with WT-CMs (Figures 5E–H), consistent with previous studies (Miller et al., 2017; El-Battrawy et al., 2019). However, R1879W-CMs exhibited sustained triggered activity (Figure 5I), which is a typical characteristic of BrS patient-specific iPSC-derived CMs (Liang et al., 2016). Taken together, these results demonstrated that these epi-BEs could evaluate and recapitulate the functions of a novel mutation identified from a patient.

### Off-Target Analysis

The epi-BEs enable long-term editing due to continued expression, which may increase undesired off-target effects. We analyzed a panel of potential off-target sites for each single cell-derived clone that was used for electrophysiological recording (Supplementary Table 3). These potential off-target sites were selected using an online tool that identifies potential off-target sites (see footnote 2) (Bae et al., 2014). These sites have at least three mismatches compared with the on-target sequences. The genomic DNA was extracted, and the potential off-target sites were PCR-amplified and subsequently sequenced by Sanger sequencing. Although cells were edited for 16 days, we did not detect any off-targets from these clones (Supplementary Figures 5–7). It is noteworthy to investigate the frequency of off-target cleavage by using whole genome sequencing in future studies.

## Discussion

hPSCs have proven to be a powerful tool for developing therapeutics and modeling disease (Lan et al., 2013; Wang et al., 2014). However, the generation of iPSCs from patients is a time-consuming and laborious process. Alternatively, mutations can be introduced into hPSCs by genome editing. Geng et al. (2020) developed a quick and efficient CRISPR/Cas9 genome editing procedure in iPSCs. We have previously demonstrated that the epiCRISPR system improves greatly the genome editing efficiency (Xie et al., 2017), but this technology can only introduce indels into cells for purposes of gene knockout. To overcome this limitation, we use the episomal vector to express base editors in this study, allowing the introduction of the pathogenic point mutations into hPSCs, which are more clinically relevant. This expanded technology further establishes the link between genome editing and hPSCs to enable future studies of diseases. Because the episomal vector can replicate in cells, there is an extended time possible for genome editing. As a result, higher editing efficiency has been achieved over time. Both heterozygous and homozygous mutations can be obtained and identified when analyzing single cells. The targeting range of ABEmax and AncBE4max is limited by the NGG PAM. To expand upon the targeting range of these tools, we can generate other versions of the episomal vector, which expresses other versions of base editors, such as SpCas9-NG- and SauriCas9-derived base editors (Nishimasu et al., 2018; Hu et al., 2020).

Our engineered hPSC-derived CMs successfully recapitulate disease phenotypes at the cellular level. We generate a total of five LQTS models, including two LQT1 models, two LQT2, models and one LQT3 model. All of these disease models display significantly prolonged APDs and FPDc, consistent with those of previous studies (Liang et al., 2013; Wang et al., 2014; Yoshinaga et al., 2019). We finally evaluated a novel mutation identified from a BrS patient. Interestingly, this mutation does not influence APD and FPDc but induces sustained triggered beats, consistent with BrS phenotypes at cellular levels when studied previously (Liang et al., 2016; Miller et al., 2017; El-Battrawy et al., 2019). In summary, these data demonstrate the utility of epi-BEs and provide the biomedical research community with a useful tool for diseasing modeling and deciphering novel mutations.

## Data Availability Statement

The datasets presented in this study can be found in online repositories. The names of the repository/repositories and accession number(s) can be found in the article/Supplementary Material.

## Ethics Statement

The studies involving human participants were reviewed and approved by Renji Hospital of Shanghai Jiao Tong University. The patients/participants provided their written informed consent to participate in this study.

## Author Contributions

All authors listed have made a substantial, direct and intellectual contribution to the work and approved it for publication.

## Conflict of Interest

The authors declare that the research was conducted in the absence of any commercial or financial relationships that could be construed as a potential conflict of interest.

## Footnotes

1 http://www.rgenome.net/cas-offinder/
