# Supplementary material for: Base Editing Mediated Generation of Point Mutations Into Human Pluripotent Stem Cells for Modeling Disease
Source: Front Cell Dev Biol. 2020 Sep 25;8:590581. doi: 10.3389/fcell.2020.590581 (PMC7546412; doi:10.3389/fcell.2020.590581)
Supplement: Supplementary file 1 [file Data_Sheet_1.docx]

**
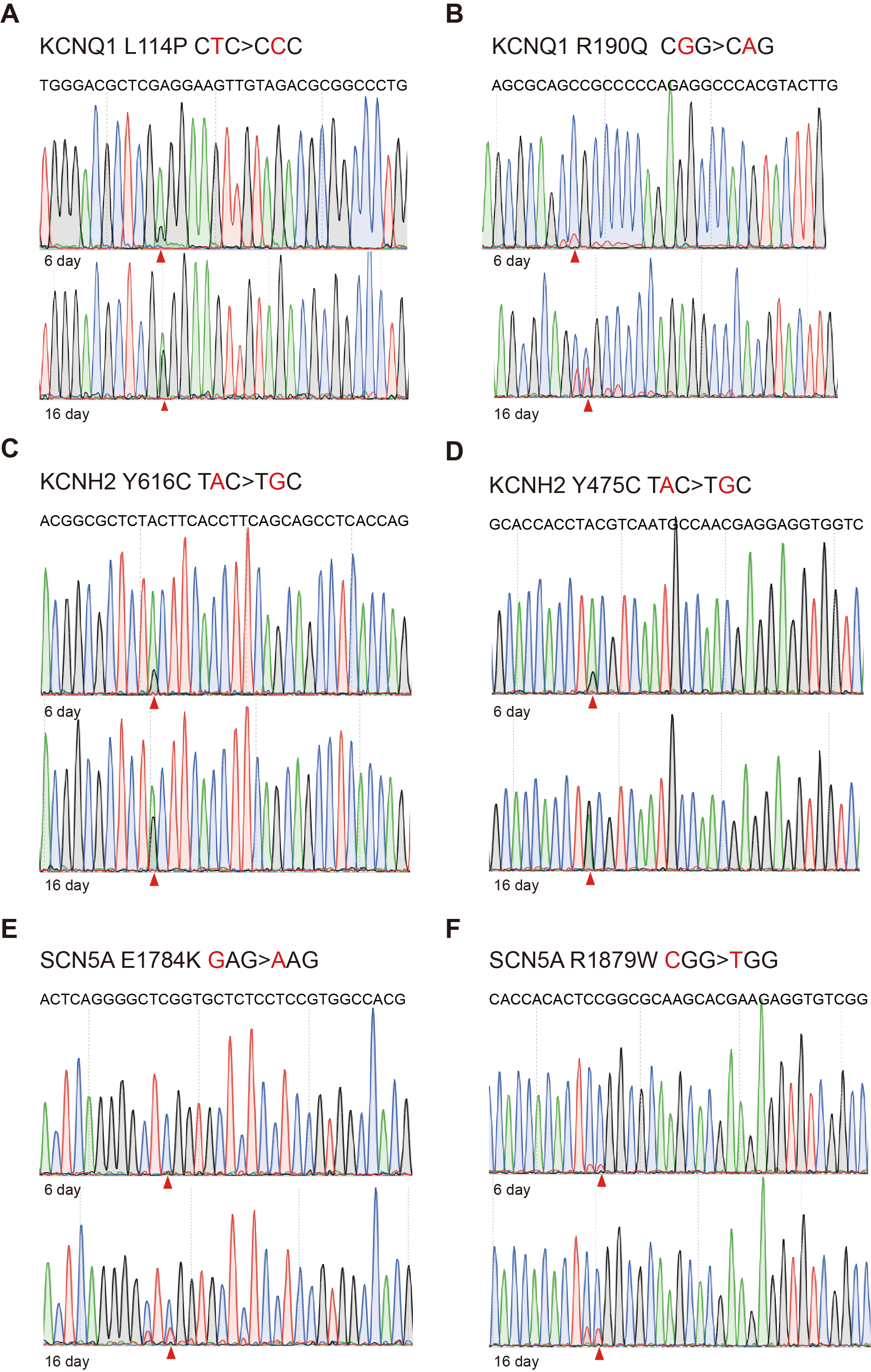
**

**Supplementary Figure 1.** Base editing efficiency on day 6 and day 16 in H9 cells. The mutated nucleotide is shown in red; the mutated nucleotide is indicated by the red triangle.

**
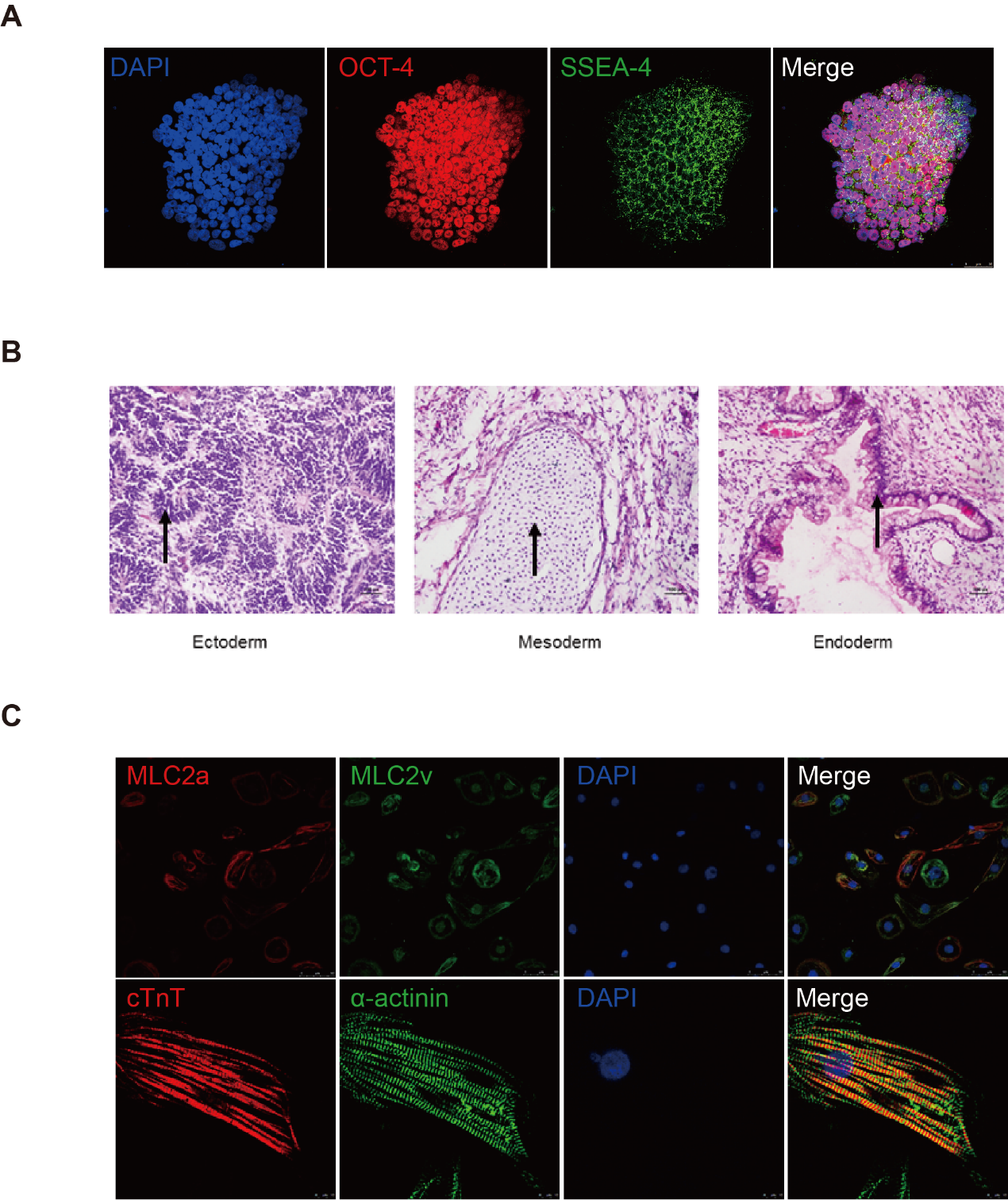
**

**Supplementary Figure 2.** Pluripotency assessment after base editing and success of cardiac differentiation. **(A)** Base editing did not influence expression of pluripotency markers OCT-4 and SSEA-4. **(B)** After base editing, the cells can differentiate into three germ layer lineages including ectoderm, endoderm and mesoderm. **(C)** All derived cardiomyocytes were stained with cardiac markers cTnT and α-actinin and ventricular/ atrial-specific marker MLC2v/a. The results showed the success of cardiac differentation.

**
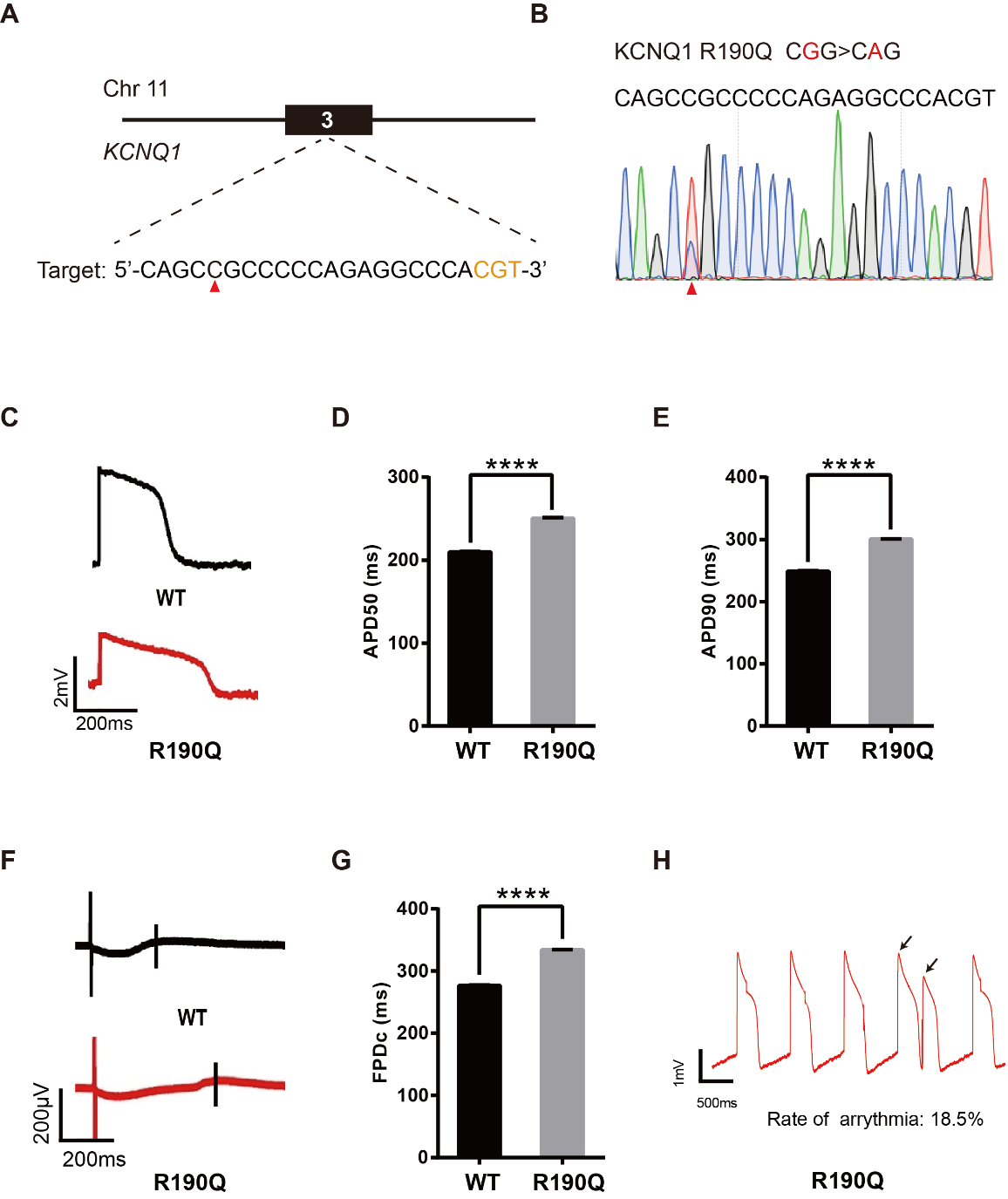
**

**Supplementary Figure 3.** Base editing of KCNQ1-R190Q for LQT1 modeling. **(A)** R190Q target site on KCNQ1. “CGT” PAM sequence is shown in orange; target nucleotide is indicated by the red triangle. **(B)** A heterozygous clone is confirmed by Sanger sequencing. The mutated nucleotide is shown in red; the mutated nucleotide is indicated by the red triangle. **(C)** Single trace of action potentials in WT-CMs and R190Q-CMs. **(D-E)** Quantification of action potential at APD50 and APD90. n= 3 independent experiments., unpaired t-test, P<0.0001. **(F)** Signals of Field Potential Duration recorded by the MEA machine. **(G)** Quantification of corrected Field Potential Durations (FPDc). n= 3 independent experiments., unpaired t-test, P<0.0001. **(H)** Respective traces of action potentials. The abnormal AP signals were labeled by black arrows.

**
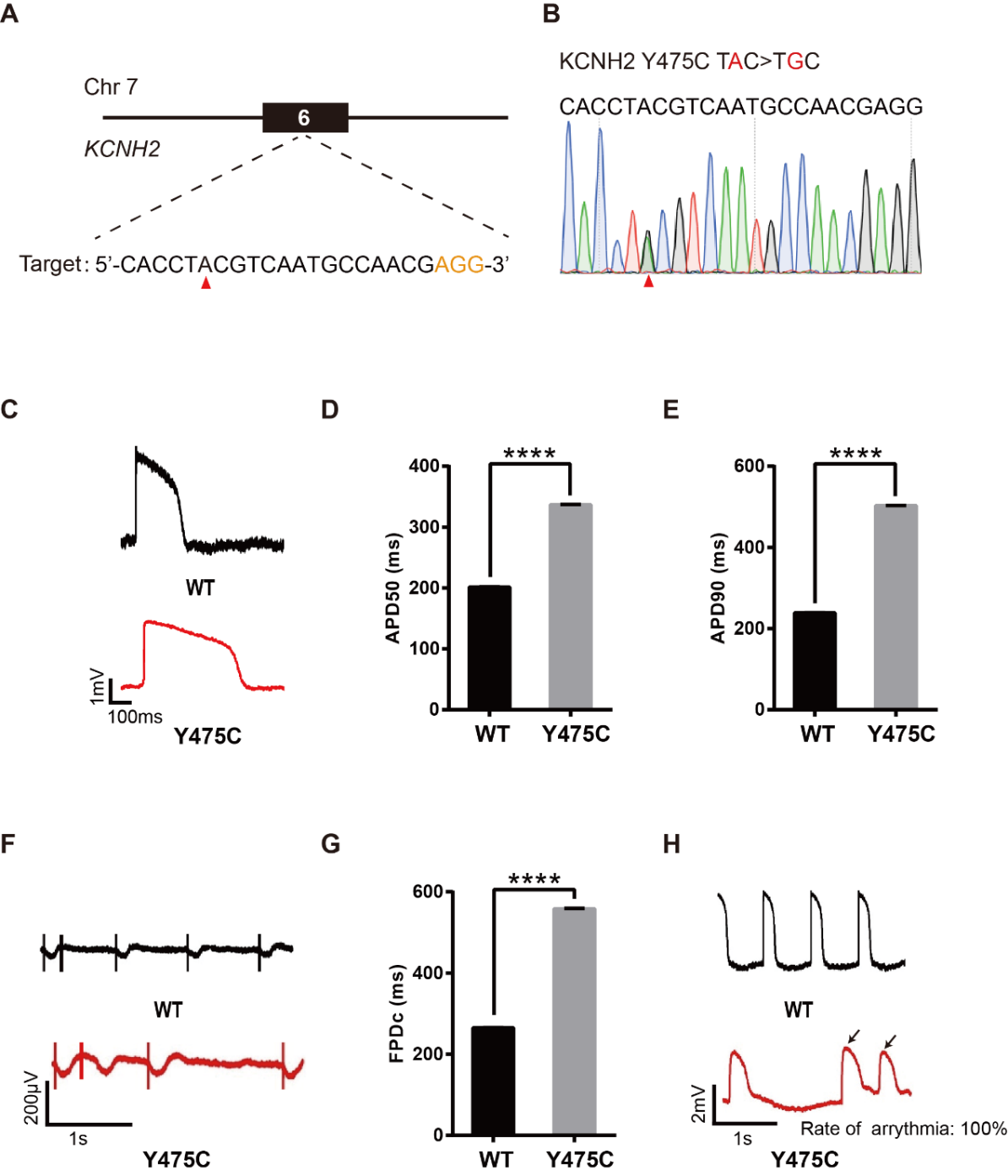
**

**Supplementary Figure 4.** Base editing of KCNH2-Y475C for LQT2 modeling. **(A)** Y475C target site on KCNH2. “AGG” PAM sequence is shown in orange; target nucleotide is indicated by the red triangle. **(B)** A heterozygous clone is confirmed by Sanger sequencing. The mutated nucleotide is shown in red; the mutated nucleotide is indicated by the red triangle. **(C)** Single trace of action potentials in WT-CMs and Y475C-CMs. **(D-E)** Quantification of action potential at APD50 and APD90. n= 3 independent experiments., unpaired t-test, P<0.0001. **(F)** Signals of Field Potential Duration recorded by the MEA machine. **(G)** Quantification of corrected Field Potential Durations (FPDc). n= 3 independent experiments., unpaired t-test, P<0.0001. **(H)** Representative traces of action potentials. The abnormal AP signals were labeled by black arrows.

**
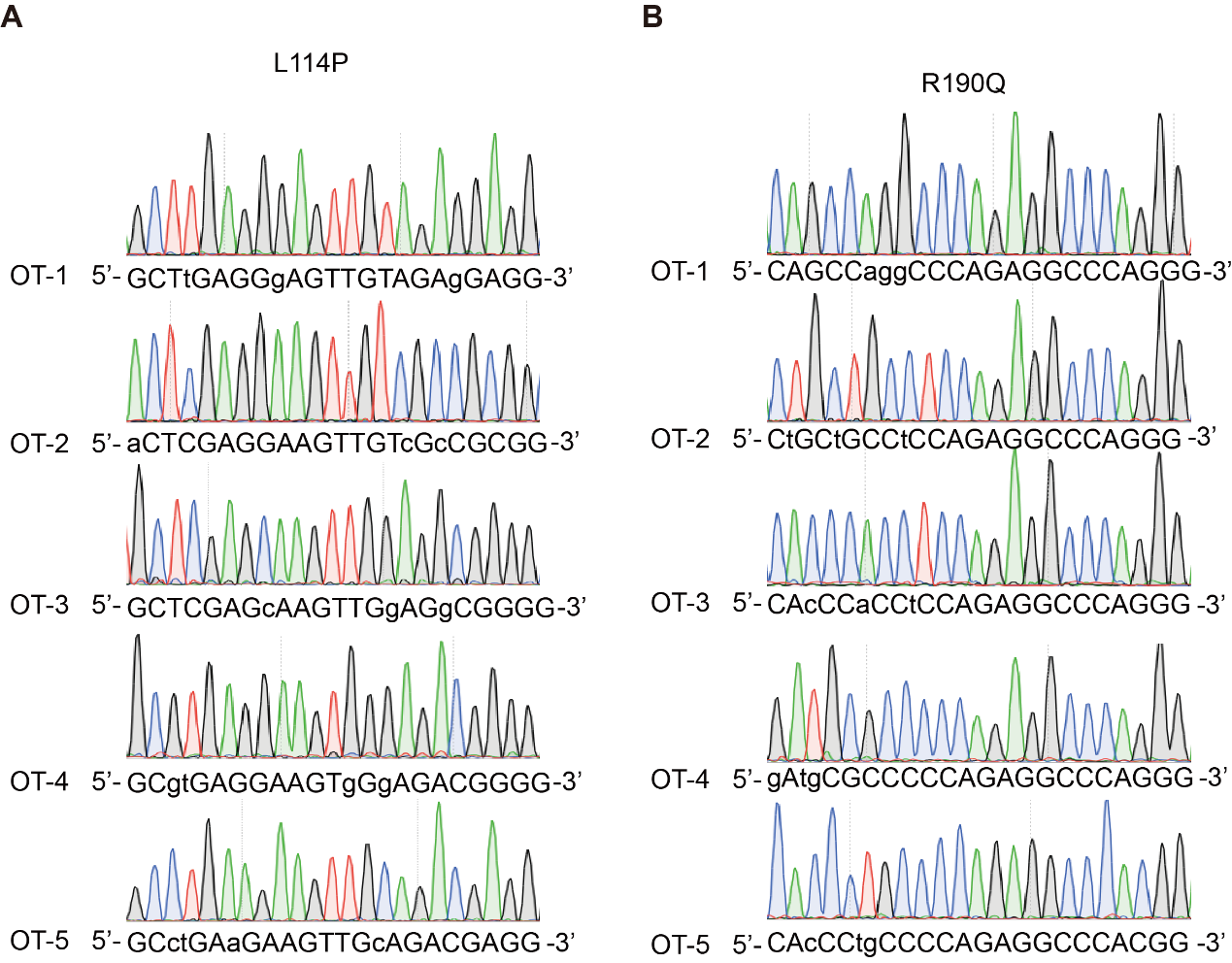
**

**Supplementary Figure 5.** Off-target analysis of the target sites on KCNQ1 in H9 cells.

**
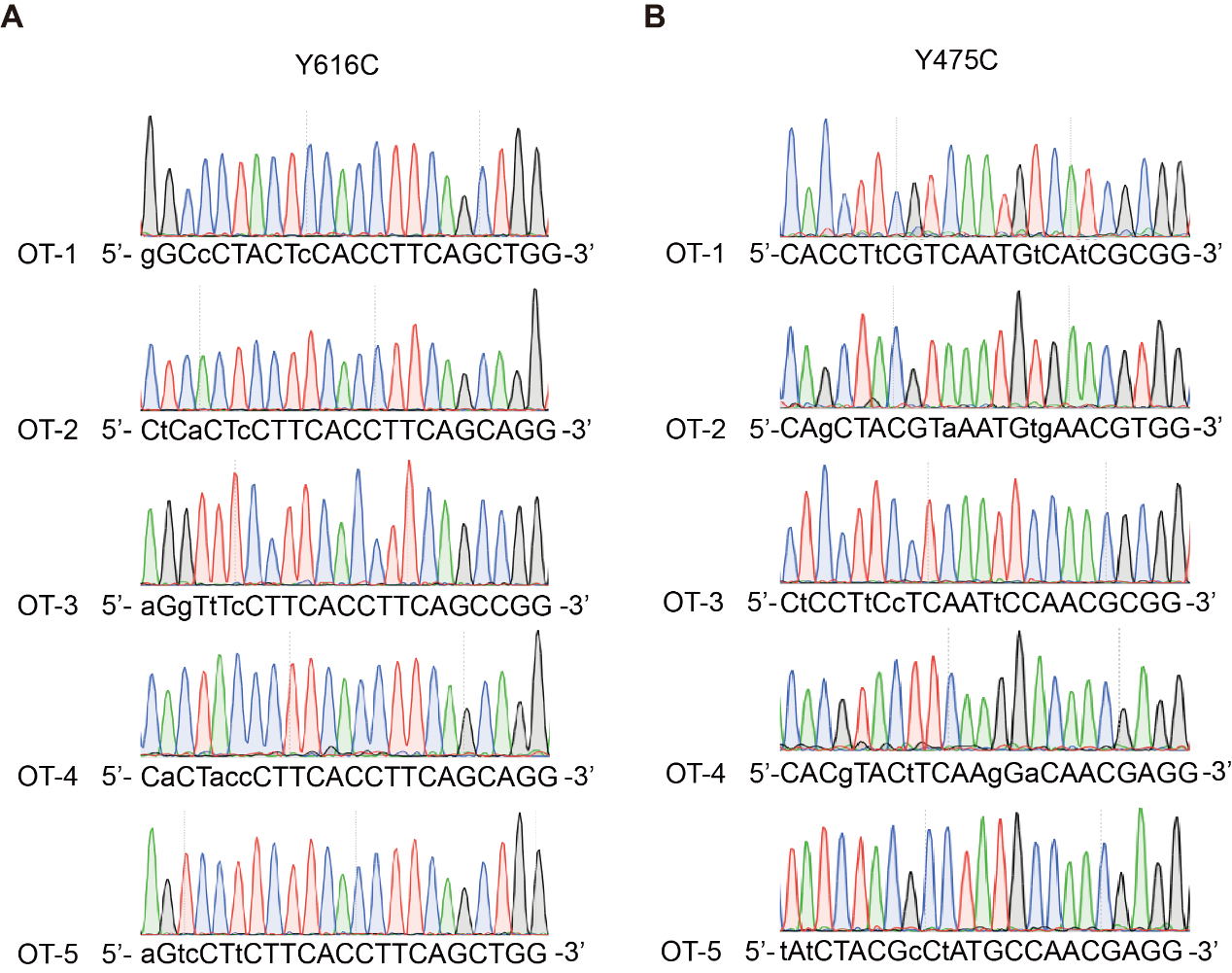
**

**Supplementary Figure 6.** Off-target analysis of the target sites on KCNH2 in H9 cells.

**
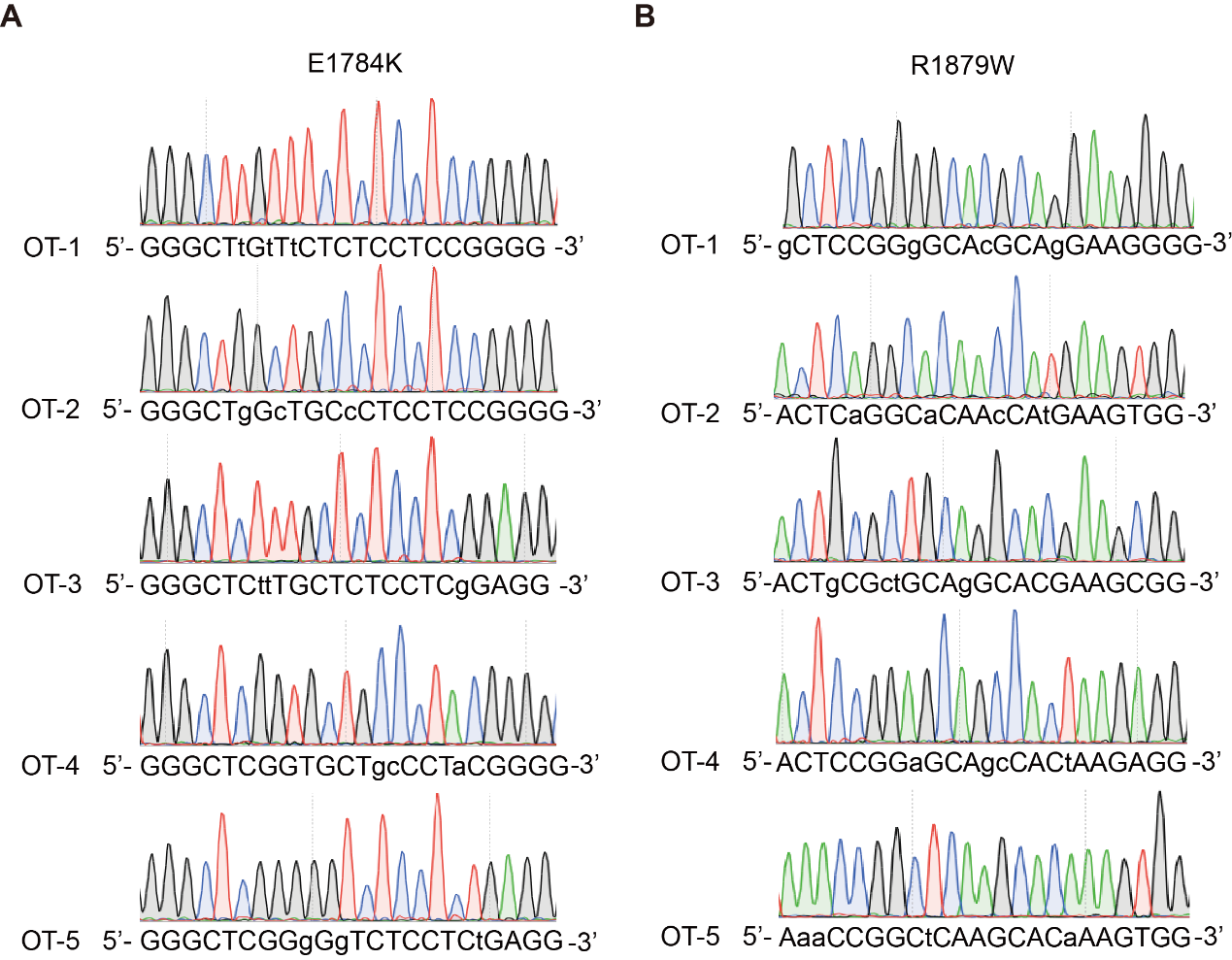
**

**Supplementary Figure 7.** Off-target analysis of the target sites on SCN5A in H9 cells.

**Supplementary Table 1. Oligo sequences**

| **Name** | **Sequence** (**5’→3’**) |
| --- | --- |
| **gRNA sequence of target site** | |
| 114-gRNA-F | tttgctcgaggaagttgtagacg |
| 114-gRNA-R | aaccgtctacaacttcctcgagc |
| 190-gRNA-F | tttgcagccgcccccagaggccca |
| 190-gRNA-R | aactgggcctctgggggcggctgc |
| 616-gRNA-F | tttgcgctctacttcaccttcagc |
| 616-gRNA-R | aacgctgaaggtgaagtagagcgc |
| 475-gRNA-F | tttgcacctacgtcaatgccaacg |
| 475-gRNA-R | aaccgttggcattgacgtaggtgc |
| 1784-gRNA-F | tttgggctcggtgctctcctccg |
| 1784-gRNA-R | aaccggaggagagcaccgagccc |
| 1879-gRNA-F | tttactccggcgcaagcacgaag |
| 1879-gRNA-R | aaccttcgtgcttgcgccggagt |
| Site1-gRNA-F | tttgatgagataatgatgagtca |
| Site1-gRNA-R | aactgactcatcattatctcatc |
| Site2-gRNA-F | tttgaagatagagaatagactgc |
| Site2-gRNA-R | aacgcagtctattctctatcttc |
| Site3-gRNA-F | tttggcccagactgagcacgtga |
| Site3-gRNA-R | aactcacgtgctcagtctgggcc |
| Site4-gRNA-F | tttggaatcccttctgcagcacc |
| Site4-gRNA-R | aacggtgctgcagaagggattcc |
| **Primers used for PCR** | |
| KCNQ1-114-F | cctggccaagaagtgcc |
| KCNQ1-114-R | gcgagaagatgcgcacc |
| KCNQ1-190-F | tcaccatccgcagcagg |
| KCNQ1-190-R | cccctacccccaacacc |
| KCNH2-616-F | atgagacacctgtgctggg |
| KCNH2-616-R | cagtagtccccgccctg |
| KCNH2-475-F | gcattacagccccttcaagg |
| KCNH2-475-R | ctgtcctcctcgccacc |
| SCN5A-1784-F | atgtcaagtgggaggctgg |
| SCN5A-1784-R | ggtgaaggcaaagagaatgtcc |
| SCN5A-1879-F | gccgatgccctgtctgag |
| SCN5A-1879-R | cgctggtggctctagtgac |
| Site1-F | cattccatgctacactatctgcc |
| Site1-R | agcctcctgagtagctgg |
| Site2-F | gtccagaccaatgtcctgg |
| Site2-R | ctcatcaataaatggtgctggg |
| Site3-F | gtcacagtggcaaatgaggc |
| Site3-R | ttcatgcaggtgctgaaagc |
| Site4-F | ggatgccctacatctgctctc |
| Site4-R | ctggaagttcgctaatcccg |
| **Primers used for plasmid construction** | |
| EBV-F | ggagacggtcacagcttg |
| EBV-R | gctgtccgtttcatggtggcggatcccgcg |
| EBV-2-F | agaagagcgatgctcttcg |
| EBV-2-R | gccggatcatctagttcgac |
| ABEmax-F | gccaccatgaaacggacagc |
| ABEmax-R | ctagaaggcacagtcgaggcttagactttcctcttcttcttgggctcg |
| AncBE4-F | gccaccatgaaacggacagc |
| AncBE4-R | ctagaaggcacagtcgaggc |
| BSD-F | agcctcgactgtgccttctag |
| BSD-R | caagctgtgaccgtctcc |
| mU6-F | gtcgaactagatgatccggc |
| mU6-R | cgaagagcatcgctcttct |
| 1111-UP-F | tgccccaagtgaatatcgtg |
| 1111-DOWN-R | cggtgatgctctggtggatc |
| epi-backbone-F | gatccaccagagcatcaccg |
| epi-backbone-R | cacgatattcacttggggca |

**Supplementary Table 2.** Summary of base editing efficiency used in this study. All results were analyzed by EditR, n= 3 independent experiments.

| **Target** | **Day** | **293T** | **HeLa** | **H9** | **iPS** |
| --- | --- | --- | --- | --- | --- |
| **Site 1** | 6 | 43.7% | 37.3% | 12.7% | 10.3% |
|  | 11 | 44% | 84.7% | 20% | 23.7% |
|  | 16 | 43% | 84% | 37% | 49.7% |
| **Site 2** | 6 | 43.3% | 46% | 15.7% | 24.7% |
|  | 11 | 45.3% | 89% | 30% | 46% |
|  | 16 | 44.7% | 89.3% | 50.7% | 59.3% |
| **Site 3** | 6 | 44% | 40.3% | 13.3% | 18% |
|  | 11 | 39.3% | 72.7% | 23.7% | 27.7% |
|  | 16 | 49.7% | 86% | 39.3% | 37.3% |
| **Site 4** | 6 | 31% | 45.7% | 11% | 8.7% |
|  | 11 | 31.3% | 81% | 28% | 25% |
|  | 16 | 42% | 88% | 33.3% | 39.3% |
| **114** | 6 | n/a | n/a | 15.3% | n/a |
|  | 16 | n/a | n/a | 34% | n/a |
| **190** | 6 | n/a | n/a | 10% | n/a |
|  | 16 | n/a | n/a | 31% | n/a |
| **616** | 6 | n/a | n/a | 15.3% | n/a |
|  | 16 | n/a | n/a | 37% | n/a |
| **475** | 6 | n/a | n/a | 18.7% | n/a |
|  | 16 | n/a | n/a | 53% | n/a |
| **1784** | 6 | n/a | n/a | 5.7% | n/a |
|  | 16 | n/a | n/a | 21% | n/a |
| **1879** | 6 | n/a | n/a | 10% | n/a |
|  | 16 | n/a | n/a | 18% | n/a |

**Supplementary Table 3.** gRNA sequence (with PAM) of potential off-target sites.

| **Target site** | **Gene** | **Sequence** | |
| --- | --- | --- | --- |
| L114P | KCNQ1 | on-target | GCTCGAGGAAGTTGTAGACGCGG |
|  |  | off-target-1 | GCTtGAGGgAGTTGTAGAgGAGG |
|  |  | off-target-2 | aCTCGAGGAAGTTGTcGcCGCGG |
|  |  | off-target-3 | GCTCGAGcAAGTTGgAGgCGGGG |
|  |  | off-target-4 | GCgtGAGGAAGTgGgAGACGGGG |
|  |  | off-target-5 | GCctGAaGAAGTTGcAGACGAGG |
| R190Q | KCNQ1 | on-target | CAGCCGCCCCCAGAGGCCCACGT |
|  |  | off-target-1 | CAGCCaggCCCAGAGGCCCAGGG |
|  |  | off-target-2 | CtGCtGCCtCCAGAGGCCCAGGG |
|  |  | off-target-3 | CAcCCaCCtCCAGAGGCCCAGGG |
|  |  | off-target-4 | gAtgCGCCCCCAGAGGCCCAGGG |
|  |  | off-target-5 | CAcCCtgCCCCAGAGGCCCACGG |
| Y616C | KCNH2 | on-target | CGCTCTACTTCACCTTCAGC |
|  |  | off-target-1 | gGCcCTACTcCACCTTCAGCTGG |
|  |  | off-target-2 | CtCaCTcCTTCACCTTCAGCAGG |
|  |  | off-target-3 | aGgTtTcCTTCACCTTCAGCCGG |
|  |  | off-target-4 | CaCTaccCTTCACCTTCAGCAGG |
|  |  | off-target-5 | aGtcCTtCTTCACCTTCAGCTGG |
| Y475C | KCNH2 | on-target | CACCTACGTCAATGCCAACGAGG |
|  |  | off-target-1 | CACCTtCGTCAATGtCAtCGCGG |
|  |  | off-target-2 | CAgCTACGTaAATGtgAACGTGG |
|  |  | off-target-3 | CtCCTtCcTCAATtCCAACGCGG |
|  |  | off-target-4 | CACgTACtTCAAgGaCAACGAGG |
|  |  | off-target-5 | tAtCTACGcCtATGCCAACGAGG |
| E1784K | SCN5A | on-target | GGGCTCGGTGCTCTCCTCCGTGG |
|  |  | off-target-1 | GGGCTtGtTtCTCTCCTCCGGGG |
|  |  | off-target-2 | GGGCTgGcTGCcCTCCTCCGGGG |
|  |  | off-target-3 | GGGCTCttTGCTCTCCTCgGAGG |
|  |  | off-target-4 | GGGCTCGGTGCTgcCCTaCGGGG |
|  |  | off-target-5 | GGGCTCGGgGgTCTCCTCtGAGG |
| R1879W | SCN5A | on-target | ACTCCGGCGCAAGCACGAAGAGG |
|  |  | off-target-1 | gCTCCGGgGCAcGCAgGAAGGGG |
|  |  | off-target-2 | ACTCaGGCaCAAcCAtGAAGTGG |
|  |  | off-target-3 | ACTgCGctGCAgGCACGAAGCGG |
|  |  | off-target-4 | ACTCCGGaGCAgcCACtAAGAGG |
|  |  | off-target-5 | AaaCCGGCtCAAGCACaAAGTGG |
